# Depression Care Management: Can Employers Purchase Improved Outcomes?

**DOI:** 10.1155/2011/942519

**Published:** 2011-06-08

**Authors:** Kathryn Rost, Donna Marshall, Benjamin Shearer, Allen J. Dietrich

**Affiliations:** ^1^Department of Mental Health Law and Policy, College of Behavioral and Community Sciences, University of South Florida, Tampa, FL 33612, USA; ^2^Colorado Business Group on Health, Denver, CO 80228, USA; ^3^Formerly of the Department of Medical Humanities and Social Sciences, Florida State University College of Medicine, Tallahassee, FL 32306, USA; ^4^Department of Community and Family Medicine, Dartmouth Medical School, Hanover, NH 03755, USA

## Abstract

Fourteen vendors are currently selling depression care management products to US employers after randomized trials demonstrate improved work outcomes. The research team interviewed 10 (71.4%) of these vendors to compare their products to four key components of interventions demonstrated to improve work outcomes. Five of 10 depression products incorporate all four key components, three of which are sold by health maintenance organizations (HMOs); however, HMOs did not deliver these components at the recommended intensity and/or duration. Only one product delivered by a disease management company delivered all four components of care at the recommended intensity and duration. This “voltage drop,” which we anticipate will increase with product implementation, suggests that every delivery system should carefully evaluate the design of its depression product before implementation for its capacity to deliver evidence-based care, repeating these evaluations as new evidence emerges.

## 1. Introduction

Recent studies estimate that 7.6% of employees suffer a major depressive episode each year [1]. Depression substantially reduces an employee's ability to work, as evidenced by increased absenteeism [2–4] and reduced productivity at work [2–7] with annual work costs approaching $24 billion (Y2K $US) [1]. As the most prevalent disorder of the principal conditions causing work loss in the American workforce, [8, 9] depression will soon become the leading cause of disability in industrialized countries [10]. 

Encouragingly, depression care management has been shown to improve both clinical and work outcomes in depressed employees. Depression care management provides systematic education, monitoring, and clinician feedback to individuals with depression. A randomized controlled trial (RCT) conducted in depressed employees of over 100 companies demonstrated that depression care management improved absenteeism and productivity at work over two years, with no differential effectiveness across the occupational spectrum [11]. A second RCT conducted in depressed employees of 16 companies demonstrated that an intervention providing depression care management improved effective work hours over one year [12]. The contribution of depression care management to improved work outcomes is further supported by Monte Carlo simulations and observational studies [13, 14]. Cost-benefit analysis demonstrates that depression care management intervention has the potential to provide employers a return on investment across a range of critical assumptions [15]. Scientists proceeded to develop a calculator that employers can use to estimate return on investment across critical assumptions [16]. The calculator provides a conservative estimate of the impact of depression care management on employer savings by using salary/benefits to assign an economic value to the impact, although leading economic theorists note that this approach consistently underestimates the losses employers absorb [17] by assuming that economic gains are realized only when work is not made up by coworkers and/or the employee and by not including employer savings from reducing turnover. 

Theoretically, employers can improve clinical outcomes for their depressed employees while reducing their own costs if (1) they purchase a product that provides the components of evidence-based care at the intensity and duration shown to improve clinical and work outcomes in effectiveness trials and, (2) these products are priced at levels that protect the employers' return on investment. The authors address the first consideration by comparing the products that employers can currently purchase from vendors to key components of the two RCTs demonstrating the ability of depression care management to improve work outcomes in “real-world” populations and other scientific research. The objective here is to compare the stated components of vendor products to key components of models shown to improve work outcomes. 

## 2. Methods

The Colorado Business Group on Health (CBGH) identified 14 vendors who sold depression care management products in 2009 by reviewing trade association membership lists, by systematic inquiries to national health care organizations, and by web searches. The sample included 6 disease management (DM) companies, 6 health plans (HP) four of which were organized as HMOs, and 2 managed behavioral healthcare (MB) organizations. When the CBGH interviewer first reached an employee at an eligible company (generally a sales professional), the interviewer explained that a brief survey on depression care management products was being conducted, confirmed that the company sold a depression product meeting the study's definition, and requested to talk to a representative familiar with key components of the depression product the company offered (e.g., the eligible respondent). The CBGH interviewer explained to the eligible respondent that a survey was being conducted for the Colorado Business Group on Health, a not-for-profit health coalition working with employers to increase value-based purchasing. The interviewer explained that CBGH would put information collected in this survey on the web to educate employers on depression care management products that provided care in accordance with best practice. Eligible respondents were not classified as nonrespondents until they failed to respond to a minimum of 20 phone calls or emails. 

In reviewing the research team's proposal for this manuscript, Florida State University's IRB determined that because subjects agreed to participate after being told that identified product descriptions were to be published on the web, the research project did not require human subjects review. In this manuscript, the team takes an additional step to protect respondents by removing all organizational identifiers except vendor type. 

The CBGH interviewer administered semistructured questions (Table 1) about four key components of the company's depression product defined from descriptions of the two depression care management models shown to improve clinical and work outcomes [11, 12]. Both models provide screening, which has been identified as a critical component of effective depression care management models in meta-analyses reviewing the international literature [18]. Both models provided protocolized education/monitoring by health care professionals with mental health backgrounds at comparable intensities; however, care management differed in duration, demonstrating improved work outcomes only as long as the intervention lasted—24 months [11] or 12 months [12]. In both models, care managers were supervised by a mental health specialist or a primary care physician working in concert with a consultation-liaison psychiatrist. In both models, care managers provided feedback to treating primary care physicians (PCPs) when patients failed to improve. These components closely overlap with components of depression care management interventions associated with improved clinical outcomes identified in meta-analysis [18, 19], see (Table 2). 

DM and MB respondents were also asked to note: (a) which populations were eligible to receive depression care management in the company's last contract (employees only, employees and spouses, children, retirees, and unemployed), (b) which of up to three characteristics the vendor most emphasizes in marketing its depression product (capacity to improve work outcomes, capacity to reduce health care costs, depression product cost, purchaser satisfaction, or patient satisfaction), and (c) descriptive data. HP respondents were not asked (a) and (b) because health plans generally offered depression products to all enrolled members. 

The research team completed the survey with 10 of 14 identified vendors (71.4%), with 4 DM vendors refusing to participate. Four of the 6 health plans were HMOs delivering health care to geographically concentrated populations, with the remaining 2 health plans delivering capitated or fee for service care to geographically dispersed populations. All 10 vendors reported they had offered depression care management products for two or more years.

**Product Components**

Five of 10 depression products incorporated four key components of depression care management shown to significantly improve work outcomes: one of 2 DM products, 0 of 2 MB products, and 4 of 6 HP products. Among the 6 HPs, 3 HMOs and 1 other HP offered a product that incorporated all four key components.

**Systematic Screening**

Depression products from all 10 vendors provided systematic screening. The primary method of screening used by products that provided depression care management to geographically disperse areas was administrative database screening while the primary method used by products serving geographically concentrated areas was office-based screening.

**Education and Monitoring**

The depression products sold by all 10 vendors provided protocolized education and short-term monitoring. Both DM products provided long-term monitoring, as did 4 of 6 HP products. Among HP products, 3 of the 4 HMOs provided both short- and long-term monitoring; however, monitoring was not provided by any of the HMOs at the level of intensity shown to improve work outcomes. Neither MB product provided long-term monitoring.

**Specialty Supervision of Care Managers**

The depression products sold by all 10 vendors provided specialty supervision of care managers.

**Care Manager Feedback to Treating PCPs**

One of 2 DM products and 1 of 2 MB products provided care manager feedback to treating PCPs. All HP products provided this component of care.

Only DM and MB vendors provided information about populations eligible to receive depression care management in the company's last contract. One DM and 1 MB vendor offered the product to employees and spouses; 1 MB vendor offered the product to employees only, and 1 DM vendor offered the product to varied populations. DM and MB vendors also provided data on characteristics the vendor most emphasized in marketing its depression. All 4 DM and MB vendors emphasized their product's capacity to improve work outcomes, with 3 of the 4 vendors reporting that they evaluated their product impact on this outcome. One DM and 1 MB vendor emphasized their product's capacity to reduce other healthcare costs and the product cost itself. Two DM vendors emphasized customer satisfaction. 

## 3. Discussion and Results

More than one-third of US healthcare spending is financed by employers who provide coverage to an estimated 90% of nonelderly individuals with private health insurance [20]. Employers who are interested in purchasing a depression product that improves work outcomes for the nearly 8% of their workforce, face considerable challenges. Given that the research community cannot agree on a definition of collaborative care [21], it should not be surprising that there is considerable variation in depression products on the U.S. market. Five of 10 vendors sell a depression product with the four key components identified in the literature; 3 of these 5 vendors were HMOs. However HMOs, like other vendors, adopted product designs that delivered education/monitoring at a lower intensity than shown to improve work outcomes. Only one of 10 products (a DM program) reported delivering the intensity and duration of all four components shown to improve work outcomes over two years [11]. 

Findings from the 4 HMOs in this study may contribute to efforts of national health care delivery systems to provide evidence-base care management for depression. Three of 4 HMOs chose a model that incorporated all four components of care; however, HMO leaders that championed these programs indicated that it was a struggle to get sufficient funding from their administrations to incorporate all components at their recommended intensity and/or duration. In response to budgetary constraints, program leaders noted they had to prioritize program resources to deliver care management to high risk cases only and/or to reduce monitoring intensity. Given the impact of the global recession on health care budgets around the world, we suspect that national health care service efforts to initiate and sustain depression care management programs will face similar pressures. 

Many U.S. employers are unaware that their current plans are not delivering high quality care to employees with depression. U.S. employers who are aware are often stymied because they purchase depression care as one part of a complex package of services, which cannot easily be altered to improve care for a given condition. Large employers, who are expected to drive initial product demand [22, 23] usually select products that provide centralized, rather than office-based, care management. The advantage of centralized models is that care managers can be trained to provide protocolized education and monitoring. The challenge of centralized models is that policy makers perceive it is difficult to provide effective feedback to PCPs located in other sites. Encouragingly, half of centralized DM and MB products provide feedback to treating PCPs. Questioned about the effectiveness of that feedback, the medical director for one DM product noted that over 90% of PCPs were willing to talk with him when he initiated a phone call; in remaining cases, he reported good success in coaching patients to request the necessary medication changes from their PCP. The disadvantage of centralized products is that the administrative database screening that these models employ cannot identify more than half of depressed employees whose PCP visits do not result in a depression diagnosis [24]. Because PCPs consistently fail to recognize the problem when their depressed patients present for care, depression screening [25, 26] has repeatedly been shown to be an important (but not stand-alone) component of depression care management programs [18]. 

We recognize the limitations of this study and its conclusions. First, studying the stated components of a depression care model produces a more encouraging picture than studying the implementation of the model. We pursued this line of inquiry when we recognized there were substantial gaps between evidence-based care and the components of care models that could only be expected to widen with implementation. Second, a more fine-grained approach is needed to characterize important components of these models such as education and monitoring. In a limited survey, the research team prioritized studying monitoring intensity over education because virtually all evidence-based depression care management models specify repeated monitoring during the acute and continuation phase of care. In contrast, because repeated monitoring directly impacts the cost of depression care management, vendors deliberately reduce monitoring intensity. Third, we acknowledge that even with concerted efforts, we were able to survey *∼*70% of eligible respondents. All 4 respondents are disease management firms, a pattern we suspect represents “nonignorable missingness.” We were surprised when one DM company whose clinical director expressed initial interest, refused to participate after administrators reviewed the survey. Fourth, there is a danger that findings from this article may be over-interpreted as providing definitive criteria for high-quality depression care management. We recognize that while the evidence for collaborative care's clinical effectiveness is better established [18, 19], the evidence for its cost-effectiveness is less so [21, 27]. While a “what we don't know” posture protects the field's scientific credibility, it clearly fails to translate “what we do know” to inform product selection decisions that employers are currently making. Thus, we argue that it is reasonable to evaluate the degree to which depression products currently on the market incorporate key components of interventions shown to improve work outcomes, with the caveat that it will be necessary to repeat this comparison on redefined criteria, particularly as additional research on cost-effectiveness emerges. Fifth, our study did not query vendors about cost and employee participation, two critical ingredients for employers to realize a potential return on investment in the purchase of these products. The CBGH calculator cited above allows employers to enter company-specific information to estimate the return on investment they can expect at given costs and employee participation rates. When we piloted this study, we quickly learned that vendors were unwilling to discuss product cost with us perhaps because they did not want product prices to be compared in public forums where it would be difficult to assure a favorable (or even fair) comparison. Future investigators are encouraged to evaluate whether depression products are priced at levels that protect the employers' return on investment and the degree to which depressed employees utilized these products once they are purchased, as both these features are critical to employers trying to purchase improved outcomes. 

In characterizing how vendors translate health care interventions tested in intervention trials to sell to purchasers, this paper documents one example of the considerable challenges in translating scientific discovery to better health care. U.S. depression care management vendors have made no cogent argument that the four components of evidence-based care are impossible to integrate into a competitively priced product that protects employer return on investment. The fact that a centrally organized disease management company sells such a product suggests that delivery systems in the U.S. and abroad can integrate evidence-based care for depression in current delivery systems.

## Figures and Tables

**Table 1:** Survey questions.

Survey questions	Criteria Addressed
(1) Does your depression care management product have a process to systematically identify employees with depression? If yes, can you describe how you identify employees with depression? If no, do you have the potential to do this if a purchaser requested it?	Criterion 1: engaging eligible patients
(2) Does your depression care management product include systematically educating patients about depression and its treatment? If no, do you have the potential to do this if a purchaser requested it?	Criterion 2a: patient education
(3) Does your depression care management program provide short-term monitoring of patients (within the first 6 months) to assess treatment adherence and symptom changes? If yes, how often does the depression care manager contact the average program participant in the first 6 months after the patient is engaged in the program? Note “as needed” if that's what the respondent says. If no, do you have the potential to do this if a purchaser requested it?	Criterion 2b: short-term monitoring
(4) Does your depression care management program provide long-term monitoring of patients (beyond 6 months) to assess treatment adherence and relapse? If yes, how often does the depression care manager contact the average program participant 7–24 months after the patient is engaged in the program? Note “as needed” if that's what respondent says. If no, do you have the potential to do this if a purchaser requested it?	Criterion 2c: long-term monitoring
(5) Does your depression care management product include regular care manager supervision by a mental health professional? If yes, how often does the supervision occur on average? If no, do you have the potential to do this if a purchaser requested it?	Criterion 3: care manager supervision
(6) Does your depression care management program include contacting the patient's primary care or other referring physician if the patient fails to improve? If yes, how often does your depression care manager contact the patient's primary care physician in an average group of 100 program participants? (a) *∼*1 in 100 program participants; (b) *∼*10 in 100; (c) *∼*20 in 100; (d) more than 20 in 100. If no, do you have the potential to do this if a purchaser requested it?	Criterion 4: PCP contact

**Table 2:** Key components of DMW products.

Components	Suggested intensity	Suggested duration
Systematic identification of eligible participants^a, b^	Once per patient	Not applicable
Protocolized education/monitoring^c^ by care manager with mental health background^a ,b^	Planned contacts 0–6 months^a^ and 7–24 months^d^	24 months^d^
Specialty supervision of care managers^a ,b^	Monthly^a^	Ongoing
Care manager feedback to treating PCPs^e^	Care manager feedback to Treating PCPs^e^	Ongoing

^a^Used by both interventions [11, 12] shown to improve work outcomes. ^b^Significant in meta-analysis [19]. ^c^Number of contacts not significant in meta-analysis [19]. ^d^Used by one intervention [11] shown to improve work outcomes. ^e^Not tested in meta-analysis. Although one of the two models offered telephonic psychotherapy as an option, it is not included as a key component because meta-analyses concluded this component did not improve clinical outcomes [19].
